# Effects of Two Kinds of Iron Nanoparticles as Reactive Oxygen Species Inducer and Scavenger on the Transcriptomic Profiles of Two Human Leukemia Cells with Different Stemness

**DOI:** 10.3390/nano10101951

**Published:** 2020-09-30

**Authors:** Tao Luo, Jinliang Gao, Na Lin, Jinke Wang

**Affiliations:** State Key Laboratory of Bioelectronics, Southeast University, Nanjing 210096, China; luotao@seu.edu.cn (T.L.); jlgao880325@163.com (J.G.); yywymail@126.com (N.L.)

**Keywords:** leukemia, iron nanoparticles, RNA-Seq, cytotoxicity

## Abstract

Leukemia is a common and lethal disease. In recent years, iron-based nanomedicines have been developed as a new ferroptosis inducer to leukemia. However, the cytotoxicity of iron nanoparticles to leukemia cells at the transcriptomic level remains unclear. This study investigated the effects of two kinds of iron nanoparticles, 2,3-Dimercaptosuccinic acid (DMSA)-coated Fe_3_O_4_ nanoparticles (FeNPs) as a reactive oxygen species (ROS) inducer and Prussian blue nanoparticles (PBNPs) as an ROS scavenger, on the transcriptomic profiles of two leukemia cells (KG1a and HL60) by RNA-Seq. As a result, 470 and 1690 differentially expressed genes (DEGs) were identified in the FeNP-treated HL60 and KG1a cells, respectively, and 2008 and 2504 DEGs were found in the PBNP-treated HL60 and KG1a cells, respectively. Among them, 14 common upregulated and 4 common downregulated DEGs were found, these genes were representative genes that play key roles in lipid metabolism (GBA and ABCA1), iron metabolism (FTL, DNM1, and TRFC), antioxidation (NQO1, GCLM, and SLC7A11), vesicle traffic (MCTP2, DNM1, STX3, and BIN2), and innate immune response (TLR6, ADGRG3, and DDX24). The gene ontology revealed that the mineral absorption pathway was significantly regulated by PBNPs in two cells, whereas the lipid metabolism and HIF-1 signaling pathways were significantly regulated by FeNPs in two cells. This study established the gene signatures of two kinds of nanoparticles in two leukemia cells, which revealed the main biological processes regulated by the two kinds of iron nanoparticles. These data shed new insights into the cytotoxicity of iron nanoparticles that differently regulate ROS in leukemia cells with variant stemness.

## 1. Introduction

Leukemia, especially acute myeloid leukemia (AML), is a lethal disease characterized by the accumulation of DNA-damaged immature myeloid precursors [1]. Only around 20% of adult cases are expected to survive past 5 years after diagnosis, and it is a leading cause of cancer death in young adults [2]. Although conventional chemotherapy is highly offensive against the bulk of leukemic cells, chemotherapy resistance in the refractory of AML is still a serious and common problem [2,3]. Thus, the development of new and specific therapeutic strategies that can overcome conventional drug resistance is still in demand.

In recent years, the development of nanomedicines as ferroptosis inducer in cancer cells has become a new promising approach to leukemia [4,5,6]. Ferroptosis is an iron-dependent, unique type of cell death due to excessive accumulation of toxic lipid reactive oxygen species (ROS) [7]. Ferroptosis can be stimulated by the GPX4 inhibitors (erastin, sorafenib, altretamine, etc.) and reagents that cause cellar iron overload (FeCl_2_, salinomycin, and hemoglobin), which leads to fueled ROS production and inhibition of tumor growth [8,9,10,11]. However, these compounds have already been challenged by the same resistance problem as that of traditional cancer drugs [12]. Iron nanoparticles are an emerging new ferroptosis inducer because it has the ability to increase iron levels and ROS. For example, a Fe_3_O_4_-based nanoparticle fabricated through self-assembly mechanism can generate ROS and can induce intracellular oxidative stress [6]. A Fenton-reaction-accelerable magnetic nanoparticle can be prepared to simultaneously enhance the local concentrations of Fe^2+^, Fe^3+^, and H_2_O_2_ to kill cancer cells [13]. Recently, a nanoparticle iron supplement, ferumoxytol, was found to have an antileukemia effect in vitro and in vivo in leukemia cells with a low level of ferroportin (FPN) by inducing ferroptosis [14].

However, as a new promising ferroptosis inducer, iron nanoparticle-induced cytotoxicity to leukemia cells at the transcriptomic level still remains unclear. This study thus investigated the effects of two kinds of iron nanoparticles, 2,3-Dimercaptosuccinic acid (DMSA)-coated Fe_3_O_4_ nanoparticles (FeNPs) and Prussian blue nanoparticles (PBNPs), on transcriptomic profiles of two leukemia cells (KG1a and HL60) by RNA-Seq. FeNPs can induce ROS generation through Fenton reactions, while PBNPs is an effective ROS scavenger with peroxidase (POD)-, catalase (CAT)-, and superoxide dismutase (SOD)-like activities [15]. HL60 is an AML cell with promyelocytic differentiation, while KG1a is a stem-like AML cell line that is resistant to chemotherapy and double negative T cell (DNT)-mediated cytotoxicity [2]. For example, fucoidan, a natural component of seaweeds with immunomodulatory and antitumor effects, was investigated in human AML cells. It can significantly increase apoptosis in HL60, but undifferentiated KG1a was resistant to the tumor inhibitory function of fucoidan [16].

## 2. Materials and Methods

### 2.1. Cell Lines and Reagents

The human acute myelogenous leukemia (AML) cell KG1a and the human acute promyelocytic leukemia (APL) cell HL60 were obtained by the China Center for Type Culture Collection (Shanghai, China). Dulbecco Modified Eagle Medium (DMEM) and fetal bovine serum (FBS) were acquired from Invitrogen Gibco (Carlsbad, CA, USA). Double antibiotics (penicillin plus streptomycin) were purchased from Beyotime Biotech (Shanghai, China). Counting Kit-8 and phosphate buffer saline were purchased from Sangon Biotech Co., Ltd. (Shanghai, China). The FeNPs and PBNPs were supplied by the Biological and Biomedical Nanotechnology Group of the State Key Lab of Bioelectronics, Southeast University (Nanjing, China) [15,17]. The size and potential of nanoparticles were measured again with a Malvern Particle size analyzer, Zetasizer Nano (Malvern Instruments, Malvern, UK).

### 2.2. Cell Viability, Iron Content, and ROS Measurement

The cell viability was measured with the CCK-8 assay (Cell Counting Kit-8; BS350B, Biosharp). The iron content was measured using a colorimetric assay. Briefly, cells were counted and then suspended in 5 M HCl. After incubation at 60 °C for 4 h, the cells were centrifuged and the supernatant was transferred. The supernatant was added with the freshly prepared detection reagent (0.08% K_2_S_2_O_8_, 8% KSCN, and 3.6% HCl) and incubated at room temperature for 10 min. The absorbance at 490 nm was measured using an absorption reader (BioTek, Winooski, VT, USA). The iron content was determined according to a standard curve generated with FeCl_3_ solution. The iron content was calculated as micrograms per cell. ROS was measured by the flow cytometer method. In brief, the cells were stained with 2′,7′-dichlorodihydrofluorescein diacetate (DCFH-DA) using Reactive Oxygen Species Assay Kit (Beyotime, Nantong, China) according to the manufacturer’s instructions. ROS changes indicated by fluorescence shift was analyzed on a CytoFLEX LX Flow Cytometer (Beckman, Brea, CA, USA). 

### 2.3. Cell Culture and Processing

The KG1a and HL60 cells were cultured in DMEM supplemented with 10% fetal bovine serum (FBS), 100 units/mL penicillin, and 100 μg/mL streptomycin. All iron nanoparticles were filtered through a 0.22-μm membrane. The KG1a and HL60 cells were exposed to 50 μg/mL of PBNPs or FeNPs for 72 h. To detect cell viability, the KG1a and HL60 cells were seeded in 96-well microplate (10^4^ cells/well). After incubation with iron nanoparticles for 72 h, 10 μL of CCK-8 solution was dropped to each well and incubated in cell culture incubator for 2 h. Finally, the absorbance at 450 nm was measured.

### 2.4. RNA-Seq Analysis

RNA sequencing (RNA-Seq) was conducted in collaboration with the Decode Genomics (Nanjing, China). Two biological replicate cell treatments were performed for each cell and nanoparticle. Totally, 12 RNA samples (two independent RNA samples of blank HL60 and KG1a, FeNP-treated HL60 and KG1a, and PBNP-treated HL60 and KG1a) were used to perform RNA-Seq analysis. The total RNA was isolated using the TRIzol^®^ Reagent Kit (Thermo Fisher Scientific, Waltham, MA, USA) following the manufacturer’s instructions. The degradation and contamination status of RNA were analyzed by 1% agarose gel electrophoresis, and the RNA purity was evaluated by Nanodrop 2000 according to the ratio of OD_260nm_/OD_280nm_ (around 1.8–2.2). The RNA concentration was accurately quantified by Qubit (≥500 ng/μL), and the insert size of the library was assessed with Agilent 2100 to judge RNA integrity. After the library quality control was qualified, Illumina sequencing was performed using a paired end 150 bp (paired end, PE150) strategy. To ensure the quality of information analysis and clean reads, the raw reads obtained by sequencing were filtered to remove dirty reads that contained adapters, excessive N (≥10%, N: bases information cannot be determined), and a large number of low-quality bases. Subsequent analysis was based on clean reads. The HISAT2 software was used to align clean reads with the reference genome.

### 2.5. GO and KEGG Analysis of Differentially Expressed Genes (DEGs)

Based on the HISAT2 results, HTSeq software was used to calculate the expression level of each mRNA gene in samples. The differentially expressed genes (DEGs, *p* < 0.05) were identified using the DESeq R package and are displayed in the Supplementary Materials File S1. DEGs with a false discovery rate (FDR) < 0.05 and absolute value of fold change ≥ 1.5 were used to perform the Gene Ontology (GO) and Kyoto Encyclopedia of Genes and Genomes (KEGG) analyses. The GO functional enrichment of DEGs was analyzed using Metascape software [18]. The signaling pathway annotations was mapped in the KEGG database. The key KEGG signaling pathway was draught by the Pathview [19].

### 2.6. RT-qPCR Analysis

The total RNA was extracted from cells using TRIzol Regent (Invitrogen, CA, USA) and purified by a treatment of DNase I (Thermo Fisher Scientific, Waltham, MA, USA). The high-quality RNA samples were used to generate the complimentary DNA (cDNA) using the PrimeScript^TM^ RT reagent Kit (TaKaRa, Japan). The PCR primers were designed by NCBI Primer BLAST (https://www.ncbi.nlm.nih.gov/tools/primer-blast/) and synthesized by Genscript Biotechnology (Nanjing, China) (Table S1). The Real-Time quantitative PCR (RT-qPCR) detection was performed using the SYBR Green master mix (Thermo Fisher Scientific, Waltham, MA, USA) according to manufacturer’s instruction. Each PCR detection was performed with three biological and technical replicates. The gene expression values were normalized to an internal control (glyceraldehyde-3-phosphate dehydrogenase, GAPDH). The relative gene expression level was calculated as the relative quantification (RQ) using the 2^–ΔΔCt^ method.

### 2.7. Statistical Analysis

All detection results were presented as mean ± standard deviation (SD). All data analysis were performed and plotted by the GraphPad Prism 8, in which the statistical significance was detected by the Student’s *t*-test. *p*-values < 0.05 were considered statistically significant.

## 3. Results and Discussion

### 3.1. Characterization of Nanoparticles and Their Effects on Cell Viability, Iron Content, and ROS

**Figure 1:**
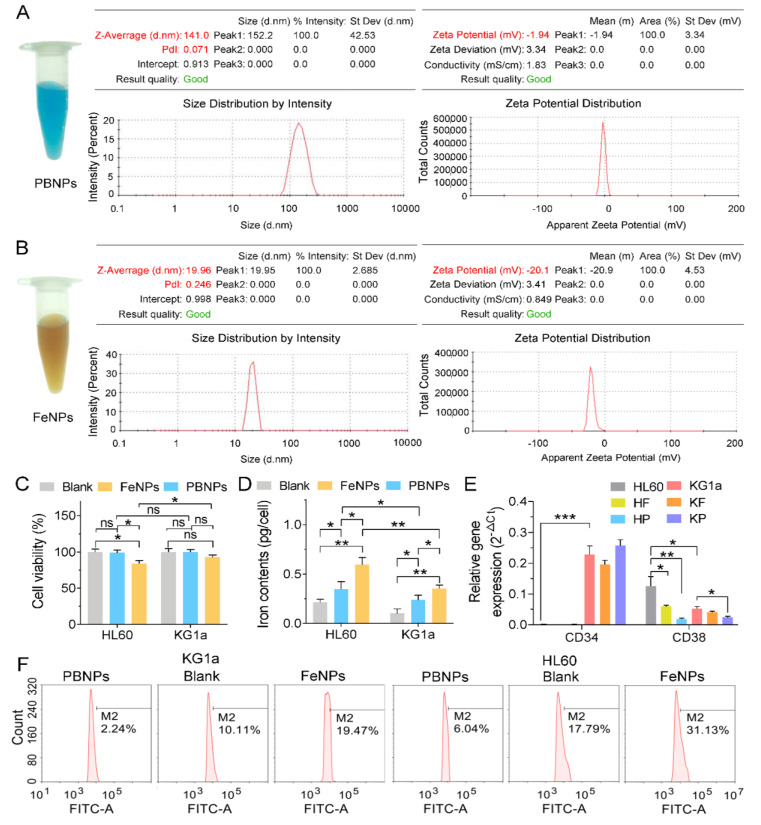
The characterization of two kinds of iron nanoparticles and their effects on KG1a and HL60: (**A**,**B**) hydrated particle size and zeta potential of Prussian blue nanoparticles (PBNPs) (**A**) and Fe_3_O_4_ nanoparticles (FeNPs) (**B**), (**C**) cell viability of the PBNP- and FeNP-treated HL60 and KG1a cells, (**D**) cellular iron contents of the PBNP- and FeNP-treated HL60 and KG1a cells, and (**E**) QPCR detection of CD34 and CD38 expression in two cells. HF, FeNP-treated HL60; HP, PBNP-treated HL60; KF, FeNP-treated KG1a; KP, PBNP-treated KG1a. ns, no significance; *, *p* < 0.05; **, *p* < 0.01; ***, *p* < 0.001. (**F**) ROS levels of the PBNP- and FeNP-treated HL60 and KG1a cells.

Two kinds of iron nanoparticles were used in this study: one was PBNPs, and the other was FeNPs. The former was in blue color, and the latter was in brown color (Figure 1A,B). The PBNPs were at sizes of 152 nm and had potentials of −1.94, while the FeNPs were at sizes of 20 nm and had potentials of −20.9 (Figure 1A,B). The CCK8 assay showed that only the cell viability of HL60 was significantly reduced by FeNP treatment (Figure 1C). The cell viability of KG1a was not significantly reduced by all treatments (Figure 1C), indicating that KG1a was more resistant to iron nanoparticles. The intracellular iron content measurement revealed that the treatment of both iron nanoparticles significantly increased the intracellular iron content of two cell lines; however, the FeNP treatment more significantly increased the intracellular iron content of two cell lines (Figure 1D). Additionally, treatment of both iron nanoparticles more significantly increased the intracellular iron content of HL60 than KG1a (Figure 1D). The qPCR detection of the expressions of two leukemia stem cell (LSC) marker genes, CD34 and CD38, indicated that CD34 was only highly expressed in KG1a and that CD38 was expressed in two cells at low levels (Figure 1E). This demonstrated that KG1a had higher stemness than HL60. When treated with two kinds of nanoparticles, CD34 expression in KG1a was slightly downregulated by FeNPs but upregulated by PBNPs, whereas CD38 was significantly downregulated by PBNPs in two cells and by FeNPs in HL60 (Figure 1E). The ROS measurement indicated that the FeNP treatment increased the ROS level in both cell lines but that the PBNP treatment decreased the ROS level in the two cell lines (Figure 1F). Altogether, these data indicated the difference between two leukemia and two kinds of iron nanoparticles.

### 3.2. RNA-Seq and de Novo Transcriptome Assembly

To investigate the transcriptome modulation that occurred during exposure to iron nanoparticles, the KG1a and HL60 cells were treated with 50 μg/mL of FeNPs and PBNPs for 72 h, respectively. To obtain reliable global gene expression profiles, RNA-Seq was performed with as many as 12 samples, which consisted of two biological replicates of each treatment. Following the removal of adapters and low-quality reads, total mapped reads, unique mapped reads, and multiple reads were summarized and are presented in Table S2. The statistics of distribution of clean reads in different regions of the genome are shown in Figure S1. After alignment with the reference genome, the statistical results of mRNA peak insert size distribution were shown in Table S3. The correlation analysis of expression levels among samples and the density map of gene expression level of all samples were further calculated to demonstrate reliability of the experiment and rationality of the sample selection (Figures S2 and S3).

### 3.3. Identification of DEGs

To discover the effects of iron nanoparticles (FeNPs and PBNPs) on the gene expression profiles of HL60 and KG1a cells, a large number of DEGs (*p* < 0.05) were identified using the FPKM (Fragments Per Kilobase of transcript per Million mapped reads) method [20]. Totally, there were 470 (260 upregulated and 210 downregulated) and 1690 (720 upregulated and 970 downregulated) DEGs in the FeNP-treated HL60 and KG1a cells, respectively, and 2008 (1015 upregulated and 993 downregulated) and 2504 (986 upregulated and 1518 downregulated) DEGs in the PBNP-treated HL60 and KG1a cells, respectively. The detailed information of these DEGs is shown in File S1 (Supplementary Materials). The results showed that KG1a had more DEGs than HL60 after the treatment of iron nanoparticles. Especially, after the treatment of FeNPs, KG1a had 3.59 times more DEGs than HL60. These data indicated that KG1a that had higher stemness than HL60 could resist iron nanoparticle-induced ferroptosis by regulating much more genes than HL60, especially encountering FeNPs, a ROS inducer. This is in agreement with the observation that FeNPs induced the significant decrease of cell viability of HL60 but had no significant effect on cell viability of KG1a (Figure 1C).

**Figure 2:**
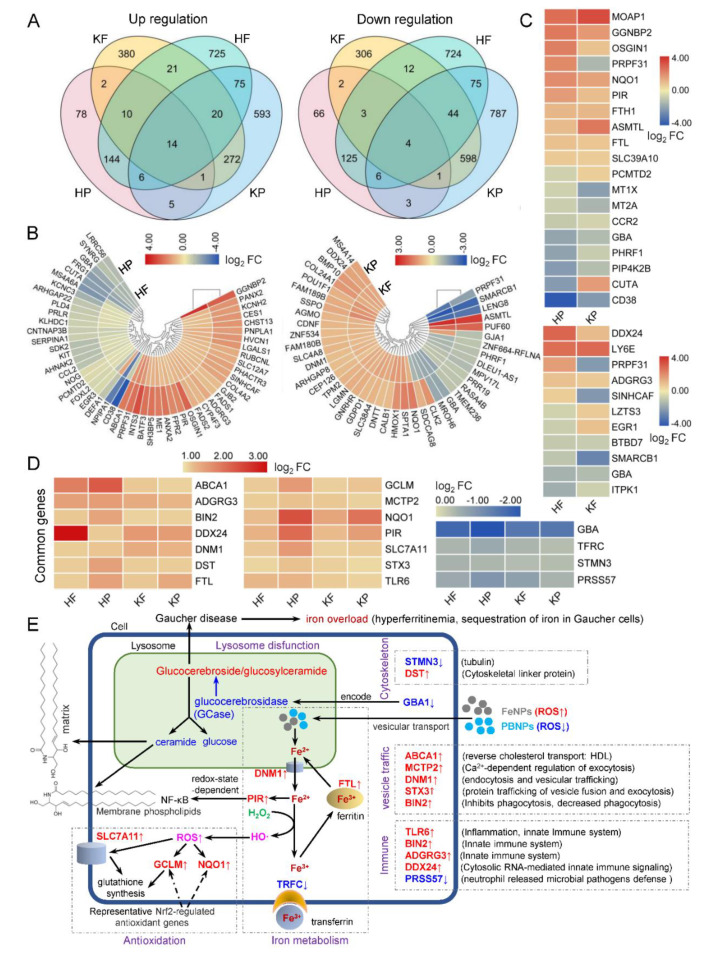
Comparisons of differentially expressed genes (DEGs) in two Leukemia cells treated with two kinds of iron nanoparticles: (**A**) number of upregulated and downregulated DEGs (fold change > 1.0) in the PBNP- and FeNP-treated HL60 and KG1a cells and their relationship, (**B**) common DEGs (fold change > 1.5) in a cell treated by two kinds of nanoparticles, (**C**) common DEGs (fold change > 1.5) in two cells treated by a nanoparticle, (**D**) common DEGs (fold change > 1.0) in two cells treated by two kinds of nanoparticles, and (**E**) schematic of cellular functions of common DEGs (fold change > 1.0) in two cells treated by two kinds of nanoparticles. The detailed information of all DEGs is shown in File S1 (Supplementary Materials). HF, FeNP-treated HL60 cells; HP, PBNP-treated HL60 cells; KF, FeNP-treated KG1a cells; and KP, PBNP-treated KG1a cells.

A four-way Venn analysis displayed the numbers of unique and common DEGs in two cells treated by two kinds of nanoparticles (Figure 2A). It was found that each cell had many unique DEGs under the treatment of two kinds of nanoparticles. Besides those unique DEGs, two kinds of nanoparticles induced some common DEGs in two cells. In comparison, two different nanoparticles induced more common DEGs in a same cell line whereas a same nanoparticle induced less common DEGs in two different cells. There were limited numbers of common DEGs in two cells treated by two kinds of nanoparticles. To provide more detailed information on common DEGs, the common genes (fold change >1.5) regulated by two kinds of nanoparticles in a same cell (Figure 2B) and a nanoparticle in two cells (Figure 2C) were identified. Totally, 52 and 42 genes were commonly regulated by two kinds of nanoparticles in HL60 and KG1a cells, respectively (Figure 2B). In contrast, only 19 genes (fold change > 1.5) were commonly regulated by PBNPs in two cells (Figure 2C) and only 11 genes (fold change > 1.5) were commonly regulated by FeNPs in two cells (Figure 2C). These genes demonstrated the obvious cell- and nanoparticle-specific features in gene expression regulation. Finally, the most important genes that were commonly regulated in two cells treated by two kinds of nanoparticles were identified (Figure 2D). It was found that 14 genes were commonly upregulated in two cells by two kinds of nanoparticles (Figure 2D) and that only 4 genes were commonly downregulated in two cells by two kinds of nanoparticles (Figure 2D). These genes should have a close relationship with the common chemical essence of two kinds of nanoparticles: iron. These genes were closely related with five biological processes, including iron metabolism, antioxidation, lipid metabolism (lysosome dysfunction), vesicle traffic (exocytosis, endocytosis, and phagocytosis), innate immune system, and cytoskeleton. These processes typically reflect what commonly happens in cells when cells are treated by iron nanoparticles no matter their modification and structure.

It was interesting that GBA was significantly downregulated in two cells by two kinds of nanoparticles (Figure 2D). GBA/GBA1 codes for glucerebrosidase (GCase), which plays a central role in the degradation of complex lipids and the turnover of cellular membranes [21]. Deficiency in GCase activity leads to accumulation of glucosylceramide/glucocerebroside in lysosome and compromised lysosomal activity, which would eventually affect lipid metabolism and trafficking [22]. GBA drives autophagy-dependent cell death [23], and the GBA mutation has a close relationship with Gaucher and Parkinson’s diseases [24]. Besides GBA1, the expression of other important lysosomal function-related genes was also changed by iron nanoparticles, such as LYST, CLN3, LAMP1, LAMP5, LAPTM5, LAPTM4A, LAPTM4B, and HPS6 in HF, HP, and KP. The compromised lysosomal activity also affects the intracellular iron metabolism because the stored iron in ferritin has to be released as Fe^2+^ in lysosome. This is coincident with the iron overload (hyperferritinemia) that occurs in Gaucher cells [25]. The ceramide produced by GCase decomposing of glucocerebroside in lysosome plays a critical role in forming membrane phospholipids and the intracellular matrix. The significant iron nanoparticle-induced downregulation of GBA1 expression thus affects membrane maintenance and repair. This effect may contribute to iron nanoparticles-induced ferroptosis. However, the molecular mechanism of how iron nanoparticles inhibit GBA1 expression still remains unclear.

GCLM and NQO1 are representative Nrf2-regulated antioxidant genes [26]. The expression of these two genes was significantly upregulated by two kinds of iron nanoparticles in two cells, suggesting that cell internalization of iron nanoparticles resulted in oxidative stress by the increase of ROS via a Fenton reaction. The cells had to upregulate these antioxidation genes and SLC7A11 to neutralize the increased ROS for maintaining redox balance. In response to the cell internalization of iron nanoparticles, cells also changed the expressions of several key iron metabolism-related genes including FTH, PIR, DNM1, and TRFC. Because iron nanoparticles were internalized into cells by phagocytosis and finally trafficked to lysosome, together with glucocerebroside accumulation in cells resulting from the iron nanoparticle-induced downregulation of the GBA1 gene, cells upregulated several important genes involved in phagocytosis, exocytosis, endocytosis, and vesicular trafficking, such as ABCA1, MCTP2, DNM1, STX3, and BIN2. The interaction of iron nanoparticles with cells also induced upregulation of several genes related to the innate immune system, such as TLR6, BIN2, ADGRG3, and DDX24. This is in agreement with a previous report that iron nanoparticles could induce virus-like immune responses [27].

**Figure 3:**
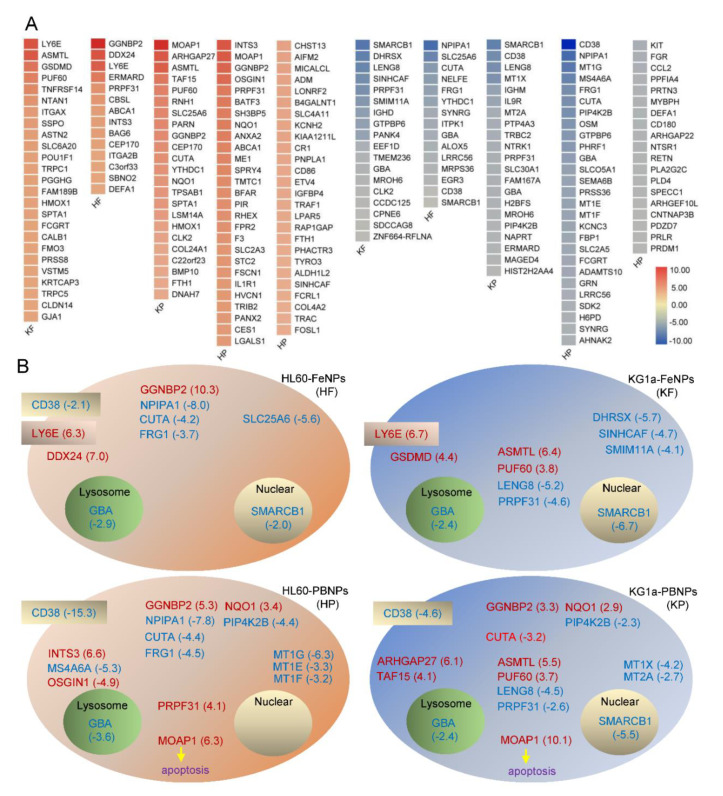
Gene signatures of two Leukemia cells treated by two kinds of iron nanoparticles: (**A**) most significantly regulated DEGs (fold change > 2.0) in two cell lines treated by two kinds of iron nanoparticles and (**B**) schematic of signature genes (fold change > 2.0) in two cell lines treated by two kinds of iron nanoparticles. The common genes were placed at the same positions in schematic cells for comparing.

To further establish a high-confidence gene signature of the two Leukemia cells treated by two kinds of iron nanoparticles, the DEGs that were most significantly regulated by the nanoparticle treatments were screened (Figure 3A). These DEGs had fold changes over 2.0. The top ones of these genes were schematically shown in cells with different treatments (Figure 3B) to indicate their distributions and relationship. It was found that GBA was the only common signature gene highly downregulated in two cells under the treatment of two kinds of iron nanoparticles. CD38 was commonly downregulated and GGNBP2 was commonly upregulated in HF, HP, and KP, respectively. SMACB1 was commonly downregulated in HF, KF, and KP. SMACB1 is a core subunit of the SWI/SNF (BAF) chromatin-remodeling complex and is well-recognized as a tumor suppressor gene, which is inactivated in aggressive cancers such as nearly all pediatric rhabdoid tumors [28,29]. These highly regulated common genes represent the typical gene signatures of various cells and nanoparticles. Besides these common genes, each cell showed some unique highly regulated genes when treated by different iron nanoparticles.

In gene signatures, it is very interesting that CD38 was significantly downregulated in HF, HP, and KP and that LY6E was highly upregulated in HF and KF (Figure 3B). CD38 is an LSC marker gene, and LSC is marked by CD34^+^CD38 ^low/−^. The above qPCR detection indicated that KG1a expressed high-level CD34 and low-level CD38 whereas HL60 expressed no CD34 and relatively high-level CD38 (Figure 1C), showing that KG1a has much higher stemness than HL60. The treatment of two kinds of nanoparticles all significantly downregulated CD38 in two leukemia cells, especially, PBNPs most significantly downregulated CD38 in HL60 (Figure 3B). These data suggested that both iron nanoparticles preferentially killed non-stemness leukemia cells, which thus increased the relative proportion of stemness leukemia cells in live cells after iron nanoparticle treatment. On the contrary, Ly6E was highly upregulated by FeNPs in two leukemia cells (Figure 3B). The human LY6 genes highly expressed in various cancers represent novel biomarkers for poor cancer prognosis, are required for cancer progression, and play an important role in immune escape [30,31,32,33,34,35]. More importantly, overexpression of certain Ly6 genes (Ly6D, Ly6E, Ly6K, and Ly6H) turned cancer cells into aggressive stem-like cells or allowed cancer cells to act like cancer stem cells [30]. In agreement with the downregulation of CD38, the significant upregulation of Ly6E in the two FeNP-treated leukemia cells also suggested that FeNPs preferentially killed non-stemness leukemia cells and thus allowed the proportion of stemness leukemia cells to increase in live cells. These data suggested the resistance of LSC to iron nanoparticle-induced cell death such as ferroptosis and nanoptosis [36].

### 3.4. GO Term Analysis of DEGs

**Figure 4:**
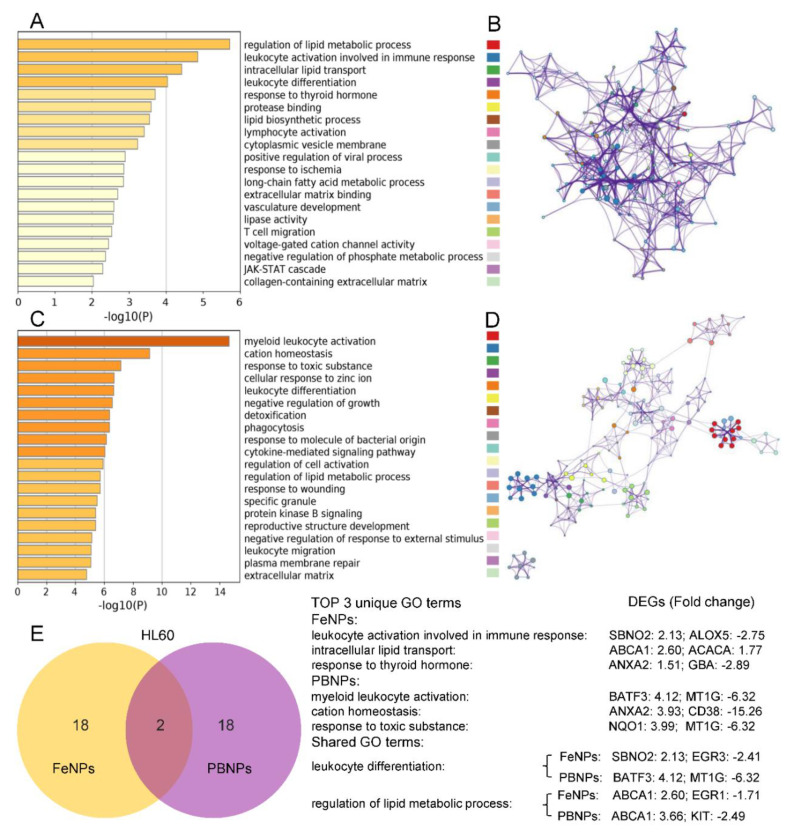
Top 20 Gene Ontology (GO) terms of the HL60 cells treated with two kinds of iron nanoparticles: (**A**,**B**) heatmap (**A**) and enrichment network (**B**) colored by the same cluster of GO terms in the FeNP-treated HL60 cells, and (**C**,**D**) heatmap (**C**) and enrichment network (**D**) colored by the same cluster of GO terms in the PBNP-treated HL60 cells. The colored labels followed the order of top 20 GO terms. (**E**) The Venn analysis of top 20 GO terms in the HL60 cells treated with PBNPs and FeNPs. The detailed information of all GO terms is shown in File S2 (Supplementary Materials).

**Figure 5:**
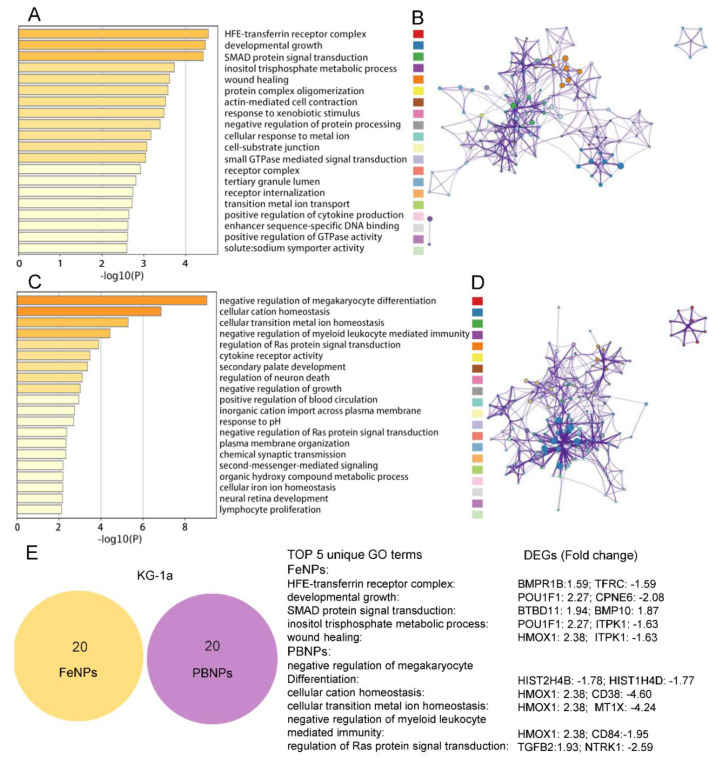
Top 20 GO terms of the KG1a cells treated with two kinds of iron nanoparticles: (**A**,**B**) heatmap (**A**) and enrichment network (**B**) colored by the same cluster of GO terms in the FeNP-treated KG1a cells, and (**C**,**D**) heatmap (**C**) and enrichment network (**D**) colored by the same cluster of GO terms in the PBNP-treated KG1a cells. The colored labels followed the order of top 20 GO terms. (**E**) Venn analysis of top 20 GO terms in the KG1a cells treated with PBNPs and FeNPs. The detailed information of all GO terms is shown in File S2 (Supplementary Materials).

To clarify the functions of the DEGs (|fold change| > 1.5, FDR < 0.05), GO function enrichment analysis was performed in Metascape (File S2 (Supplementary Materials)) [18]. The top 20 GO terms of four groups (HF: HL60-FeNPs, HP: HL60-PBNPs, KF: KG1a-FeNPs, and KP: KG1a-PBNPs) are displayed in Figure 4 and Figure 5, respectively. Because PBNPs can effectively scavenge ROS via multienzyme-like activity including peroxidase (POD), catalase (CAT), and superoxide dismutase (SOD) activity while FeNPs produced hydroxyl radicals (·OH) through the Fenton reaction and peroxidized lipids [15,37], the difference of GO terms between these two kinds of iron nanoparticles in one cell line (HL60 or KG1a) was firstly characterized. The most significant GO term in HF, HP, KF, and KP was the regulation of the lipid metabolic process, myeloid leukocyte activation, the HFE–transferrin receptor complex, and negative regulation of megakaryocyte, respectively. Interestingly, 25% of GO terms of HF was mainly enriched in “lipids”, including regulation of lipid metabolic process, intracellular lipid transport, lipid biosynthetic process, cytoplasmic vesicle membrane, long-chain fatty acid metabolic process, and lipase activity (Figure 4A,B). In comparison, only 10% of GO terms of HP were related to “lipids”, including regulation of lipid metabolic process and plasma membrane repair (Figure 4C,D). The results showed that the capability of FeNPs to produce ROS made it easier for FeNPs to regulate lipid metabolism than PBNPs. To further show the roles of genes in lipid metabolism, the DEGs in these GO terms are listed in Table 1. There were 28 (20 up- and 8 downregulated) genes in HF and 13 (9 up- and 4 downregulated) genes in HP involved in lipids regulation, of which 6 shared genes (ABCA1, FPR2, KIT, FADS1, ME1, and AHNAK2) were found. Particularly, the expression of ABCA1 was most significantly upregulated in lipid metabolism of HF and HP. ABCA1 was an important membrane-associated protein and actively participated to phosphatidylcholine, phosphatidylserine, and sphingomyelin transfer [38]. However, ABCA1 and 5 other shared genes were not found in the lipid-associated GO terms of KG1a. Besides these shared genes, ACACA was the unique gene that frequently appeared in lipid metabolism-related GO terms of HF. ACACA, acetyl-CoA carboxylase, was the first and rate-limiting step of de novo fatty acid biosynthesis [39]. This gene was also upregulated in HF and HP. This kind of significant effect of FeNPs on lipid metabolism was also found in the HepG2 cell treated by Fe_3_O_4_ nanoparticles in a previous study [36]. This previous study and this study all revealed that the treatment of Fe_3_O_4_ nanoparticles induced lipid accumulation in cells. In consistence with the previous study [36], this study also found that several lipid synthesis-related genes were upregulated in HF, including FDFT1, ACAT2, and HMGCS1. In contrast, the most significant biological process was myeloid leukocyte activation and cation homeostasis in the PBNP-treated HL60 cells (Figure 4C,D).

For KG1a, there was few GO terms related to lipid metabolism. Only three genes (ITPK1, PLCG2, and POU1F1) were involved in inositol triphosphate metabolic process in KF, and four genes (SPTA1, TGFB2, CXCR4, and EHD2) mediated plasma membrane organization in KP. In contrast, GO terms that responded to metal ions were highly enriched in KG1a (Figure 5A–D). As listed in Table 2, 4 GO terms (containing 13 genes) were found in KF and just one term (containing 4 genes) was found in HF. It implied that KG1a was more sensitive to metal ions than HL60 under the FeNP treatment. For example, TFRC and TFR2 genes were over-suppressed in KF, which belong to the transferrin receptor-like family and are necessary for cellular iron uptake [40]. Another receptor, BMPR1B, overexpressed in KF, can specifically bind the bone morphogenetic protein (BMP) to regulate a wide range of biological processes including iron homeostasis, fat and bone development, and ovulation [41].

It was found that the antioxidation-related genes were highly regulated in two cells treated by two kinds of iron nanoparticles. NQO1 and GCLM were commonly upregulated in two cells treated by two kinds of nanoparticles (Figure 2D). Two genes (NQO1 and HMOX1) that was closely associated with antioxidant metabolism were in upregulated by two kinds of nanoparticles in KG1a (File S1 (Supplementary Materials)). NQO1 and HMOX1 are representative NRF2-regulated antioxidation genes [26]. NQO1 reduces quinone to hydroquinone, and HMOX1 catalyzes the degradation of heme to biliverdin, CO, and Fe^+^. If either one of the two genes was knocked out, it would enhance erastin- and sorafenib-induced ferroptosis in hepatocellular carcinoma cells [7]. The two genes may help KG1a cells to scavenge ROS and thus resist iron nanoparticle-induced ferroptosis. Another antioxidation gene, GPX3, was only upregulated in HF. Together with the upregulated GCLM and GCLC in HF, these three typical NRF2-regulated antioxidation genes [26] revealed high oxidative stress in HF, which agrees with the decrease of cell viability of only HF in all treatments (Figure 1C). In addition, iron nanoparticles-induced oxidative stress was also demonstrated by the wide regulation of the expression of as many as 15 (HF), 42 (KF), 50 (HP), and 70 (KP) genes coding oxidase, reductase, peroxidase, dehydrogenase, epoxidase, and oxidoreductase (File S4 (Supplementary Materials)). It was interesting that a recently identified anti-ferroptosis gene, AIFM2 (renamed as ferroptosis suppressor protein 1, FSP1, a CoQ oxidoreductase) [42,43], was also upregulated in KF and HP. Many proteins sequester transition metals or transport them and thus indirectly act as antioxidants by suppressing formation of HO· from H_2_O_2_ by Fenton chemistry [44]. These proteins include ferritin (comprising light FTL1 and heavy FTH1 subunits), ferroportin (FPN1/SLC40A1), metallothionein, and ceruloplasmin. FTL was commonly upregulated by two kinds of nanoparticles in two cells (Figure 2D). FTH1 was upregulated in KF, HP, and KP (File S1 (Supplementary Materials)). Another an important antioxidation gene, SLC7A11, was commonly significantly upregulated in two cells treated by two kinds of nanoparticles (Figure 2D). SLC7A11 imported cysteine into cells for glutathione synthesis and thus plays key role in antioxidation of cells. Besides SLC7A11, as many as 13, 40, 48, and 56 soluble carrier (SLC) family genes were differentially regulated in HF, HP, KF, and KP, respectively (File S1 (Supplementary Materials)). These SLC family genes encoding passive transporters, ion coupled transporters, and exchangers were crucial for intracellular ion homeostasis [45,46]. It was worthy to note that metallothioneins (MTs) were generally downregulated in HL60 and KG1a exposed to PBNPs, including MT1E, MT1F, MT1G, MT1X, and MT2A in HL60, and MT1X and MT2A in KG1a. With a high content of cysteine residues, metallothioneins bound various heavy metals for detoxification in cells and acted as antioxidants to protect against hydroxyl free radicals [47]. The inhibited metallothioneins further supported the low metal toxicity and good antioxidant capacity of PBNPs in HL60 and KG1a. When exposed to PBNPs, this iron nanoparticle can effectively scavenge ROS via multienzyme-like activity to reduce the burden of cellular antioxidants.

### 3.5. Pathway Analysis of DEGs

**Figure 6:**
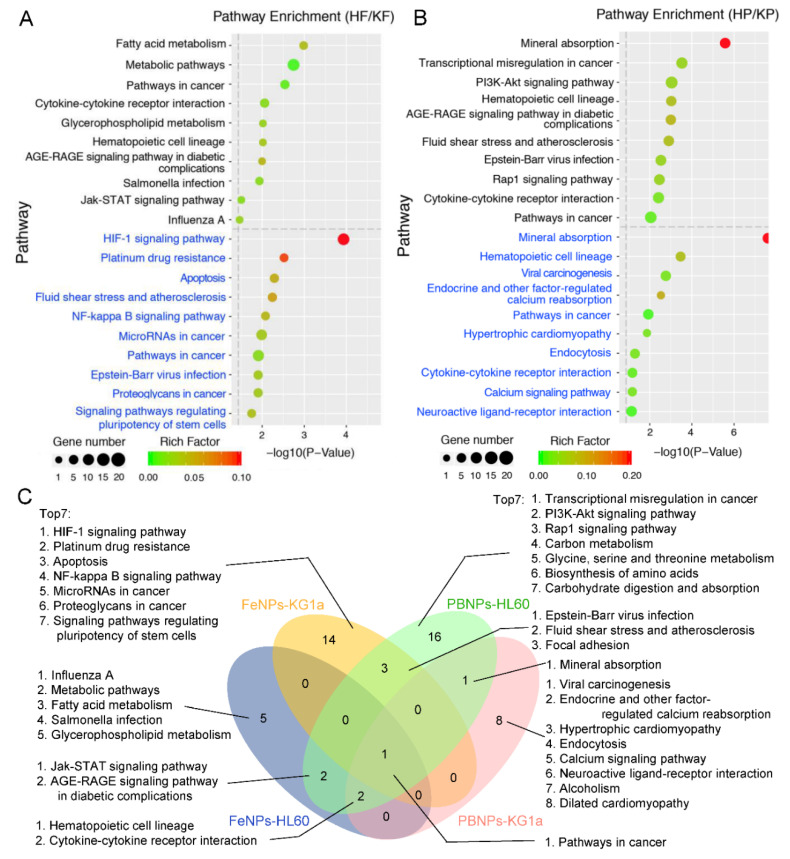
Comparative Kyoto Encyclopedia of Genes and Genomes (KEGG) pathway enrichment analysis of the DEGs (*p* < 0.05): (**A**) comparative top 10 enriched pathways of HL60 and KG1a treated with FeNPs and (**B**) comparative top 10 enriched pathways of HL60 and KG1a treated with PBNPs. The pathways in black and blue were HL60 and KG1a cells, respectively. (**C**) Four-way Venn analysis of all KEGG pathways in four groups. The detailed information of all KEGG pathways is shown in File S3 (Supplementary Materials). HF, FeNP-treated HL60 cells; HP, PBNP-treated HL60 cells; KF, FeNP-treated KG1a cells; and KP, PBNP-treated KG1a cells.

To find the pathways regulated by iron nanoparticles, the DEGs with over 1.5-fold change were annotated by the KEGG database. As a result, 106 DEGs in HF, 283 DEGs in HP, 199 DEGs in KF, and 152 DEGs in KP were enriched in 10, 25, 18, and 12 KEGG pathways, respectively (File S3 (Supplementary Materials)). The comparison of the top 10 pathways revealed that there was great difference between HF and KF, but some similarity between HP and KP (Figure 6A,B). Only the pathways in cancer were enriched in four treatments (Figure 6C).

In HF, the fatty acid metabolism was the most significantly enriched pathway, in which the ACACA, FADS1, and FADS2 genes play important roles in cell membrane formation and repair. The metabolic pathways mainly involved in lipid metabolism and glycerophospholipid metabolism were also enriched in HF. In contrast, the HIF-1 signaling way was most prominently enriched in KF, which consisted of CDKN1A, FLT1, HMOX1, IGF1R, PLCG2, TFRC, STAT3, LDHA, ENO1, PGK1, and ELOB genes (Figure S4). This signaling way can mediate adaptive responses to reduced oxygen availability; it was crucial for angiogenesis, vascular reactivity and remodeling, glucose and energy metabolism, inflammation, tumor, and iron homeostasis [48]. The hypoxia and free radicals can activate the function of HIF-1 [49]. By producing ROS, the HIF-1 signaling way was activated by FeNPs in KG1a. PLCG2 was induced by ROS to affect the IP3/DAG pathways, the ubiquitination of HIF-1α was inhibited by the downregulated ELOB, and the activity of HIF-1α was enhanced by the upregulated receptor tyrosine kinase (RTK). The iron deprivation can stimulate TFRC transcription through HIF-1 [50]. LY6E, highly upregulated in HF and KF (Figure 3B), was identified as an activator of HIF-1 and functioned as a novel conductor of tumor growth through its modulation of the PTEN/PI3K/Akt/HIF-1 axis [35].

It is interesting that the Platinum drug resistance pathway was also highly enriched in KF, in which several genes involved in platinum drug resistance were upregulated, including BIRC3, CDKN1A, GSTM2, and BBC3 (File S1 (Supplementary Materials)). ROS increase plays a key role in both platinum and iron nanoparticle-induced cancer cell death. Therefore, the activation of the platinum drug resistance pathway in KF may underline the resistance of KG1a cell to FeNP treatment (Figure 1C). Interestingly, CDKN1A and GSTM2 were also upregulated in HP and BIRC3 and CDKN1A were also upregulated in KP (File S1 (Supplementary Materials)). However, none of these genes were regulated in HF. This contributed to the decrease of cell viability of HF and no significant decrease of cell viability of HP, KF, and KP (Figure 1C).

When exposed to PBNPs, 40% top 10 pathways were identical between KG1a and HL60, in which the mineral absorption was most significantly enriched in two cells (Figure 6B). In HP and KP, Ferritin (FTH1 and FTL) and MTs were changed to regulate cellular metal ions concentration due to increased iron ions (File S3 and Figure S5 (Supplementary Materials)). In contrast, the mineral absorption pathway in KG1a was more complex (File S3 and Figure S5 (Supplementary Materials)), including the multi-responses of HMOX1 to Fe^2+^, SLC8A1(NCX1) to Na^+^ and Ca^2+^, and SLC30A1(ZNT1) to Zn^2+^ [7,51,52]. The activation of the mineral absorption pathway made the two cells survive under the treatment of PBNPs (Figure 1C); however, the significant change of lipid metabolism induced by FeNPs made HL60 easy to kill (Figure 1C) although FTL was upregulated and TFRC was downregulated (Figure 2).

### 3.6. Validation of RNA-Seq Gene Expression Levels Using RT-qPCR

**Figure 7:**
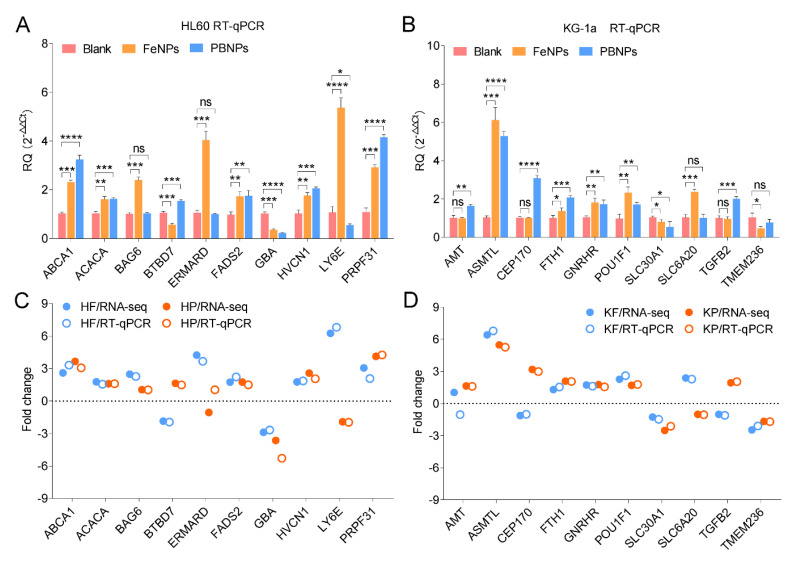
Validation of RNA-Seq DEGs using RT-qPCR: (**A**,**B**) the expression levels of 10 selected DEGs in the iron nanoparticle-treated HL60 (**A**) and KG1a (**B**) cells. All values are mean ± SD with *n* = 3. ns, no significance; *, *p* < 0.05; **, *p* < 0.01, ***, *p* < 0.001, ****, and *p* < 0.0001. (**C**,**D**) The comparison of fold change detected by RT-qPCR and RNA-Seq in the iron nanoparticle-treated HL60 and (**C**,**D**) KG1a cells.

To validate the accuracy of RNA-Seq results, ten genes in HL60 and KG1a were also detected by RT-qPCR. As a result, the RNA-Seq-detected expression regulation of 20 genes were verified by RT-qPCR detection (Figure 7A,B). Additionally, the qPCR-detected CD38 expression (Figure 1E) was also in agreement with that detected by RNA-Seq (Figure 3B). These data indicated that the RNA-Seq results were reliable.

## 4. Conclusions

This study provided the transcriptomic profiles of two leukemia cells (KG1a and HL60) that had different stemness treated by two kinds of iron nanoparticles (FeNPs and PBNPs) that had different ROS regulation capabilities. The results indicated that the expression of many genes was significantly regulated. More genes were regulated by PBNPs than FeNPs. The unique and common genes were determined. The gene signatures were established. The common genes in all treatments were closely related with iron metabolism, antioxidation, lipid metabolism, vesicle traffic, innate immune system, and cytoskeleton. The mineral absorption pathway was most significantly regulated by PBNPs in both cells, whereas the lipid metabolism pathway was most significantly regulated by FeNPs in HL60. This study shed new insights into the cytotoxicity at the gene transcription level of iron nanoparticles that differently regulate ROS in leukemia cells with different stemness. This study demonstrated why the leukemia cell with low stemness is sensitive and with high stemness is resistant to FeNPs as a ROS inducer. This study also suggested the potential resistance of stemness leukemia cells to iron nanoparticles as a ferroptosis inducer.

## Figures and Tables

**Table 1:** GO terms of lipid metabolisms in KG1a and HL60 exposed to FeNPs or PBNPs (Top 20, *p* < 0.01).

Group	Category	GO Term	LogP	NO.	Gene Symbol
HF	BP	GO:0019216~regulation of lipid metabolic process	−5.71	11	ABCA1, ACACA, CHRM5, EGR1, FPR2, KIT, FADS1, ME1, SOCS7, LPCAT1, RUBCNL
	BP	GO:0032365~intracellular lipid transport	−4.42	4	ABCA1, ACACA, ANXA2, CES1
	BP	GO:0008610~lipid biosynthetic process	−3.55	10	ACACA, ALOX5, CES1, EGR1, FPR2, FADS1, PRLR, FADS2, LPCAT1, MBOAT2
	CC	GO:0030659~cytoplasmic vesicle membrane	−3.23	11	CD38, FPR2, IFNGR2, ITGA2B, TGFA, SH3BP5, SYNRG, LPCAT1, HVCN1, AHNAK2, ADGRG3
	MF	GO:0016298~lipase activity	−2.57	4	CES1, CHRM5, PLD4, PNPLA1
HP	BP	GO:0019216~regulation of lipid metabolic process	−5.52	9	ABCA1, ADM, FGR, FPR2, KIT, FADS1, ME1, PPARG, SMARCD3
	BP	GO:0001778~plasma membrane repair	−5.07	4	DYSF, SYT11, MYOF, AHNAK2
KF	BP	GO:0032957~inositol trisphosphate metabolic process	−2.92	3	ITPK1, PLCG2, POU1F1
KP	BP	GO:0007009~plasma membrane organization	−2.35	4	SPTA1, TGFB2, CXCR4, EHD2

Note: HF: HL60-FeNPs, HP: HL60-PBNPs, KF: KG1a-FeNPs, KP: KG1a-PBNPs.

**Table 2:** GO terms of metal ion metabolisms in KG1a and HL60 exposed to FeNPs or PBNPs (Top 20, *p* < 0.01).

Groups	Category	GO Terms	LogP	NO.	Gene Symbol
HF	MF	GO:0022843~voltage-gated cation channel activity	−2.45	4	ANXA2, KCNC3, KCNH2, HVCN1
HP	BP	GO:0055080~cation homeostasis	−9.14	31	ADM, ANXA2, ATP6V0A1, CD38, ELANE, FPR2, FTH1, FTL, FYN, GATA2, GRN, GSTM2, KCNH2, MT1E, MT1F, MT1G, MT1X, MT2A, NTSR1, PLCB2, TFRC, STC2, SLC12A7, NCS1, SLC7A8, MCUB, JPH1, SLC39A10, SLC4A11, SLC24A4, CCR2
	BP	GO:0071294~cellular response to zinc ion	−6.68	6	MT1E, MT1F, MT1G, MT1X, MT2A, HVCN1
KF	CC	GO:1990712~HFE-transferrin receptor complex	−4.54	3	BMPR1B, TFR2, TFRC
	BP	GO:0071248~cellular response to metal ion	−3.17	6	NQO1, HMOX1, LGMN, TFR2, CPNE6, RASA4B
	BP	GO:0000041~transition metal ion transport	−2.72	4	TFR2, TFRC, TRPC1, TRPC5
	MF	GO:0015370~solute sodium symporter activity	−2.60	3	SLC22A1, SLC4A8, SLC6A20
KP	BP	GO:0030003~cellular cation homeostasis	−6.87	18	CALB1, CD38, FTH1, FTL, GJA1, LPAR4, HMOX1, MT1X, MT2A, PTGER3, SLC8A1, SLC30A1, CXCR4, F2RL3, SLC4A8, SLC39A10, ROGDI, CCR2
	BP	GO:0046916~cellular transition metal ion homeostasis	−5.29	7	FTH1, FTL, HMOX1, MT1X, MT2A, SLC30A1, SLC39A10
	BP	GO:0098659~inorganic cation import across plasma membrane	−2.75	4	SLC8A1, SLC30A1, SLC39A10, SLC12A8
	BP	GO:0006879~cellular iron ion homeostasis	−2.19	3	FTH1, FTL, HMOX1

Note: HF: HL60-FeNPs, HP: HL60-PBNPs, KF: KG1a-FeNPs, KP: KG1a-PBNPs.

## Supplementary Materials

The following are available online at https://www.mdpi.com/2079-4991/10/10/1951/s1, Table S1. The qPCR primers sequences used in this study; Table S2. Mapping statistics of RNA-Seq (each sample had two biological replicates); Table S3. The mRNA peak insert size in all samples; Figure S1. Statistics of the distribution of reads in different regions of the genome; Figure S2. Correlation analysis of gene expression in 12 samples; Figure S3. Density map of gene expression levels of all samples; Figure S4. KEGG analysis of the HIF-1 signaling pathway in Pathview; Figure S5. KEGG analysis of the mineral absorption pathway in Pathview; File S1. DEGs; File S2. GO terms; File S3. KEGG pathways; File S4. Redox-related DEGs.
