# Supplementary material for: Effects of Two Kinds of Iron Nanoparticles as Reactive Oxygen Species Inducer and Scavenger on the Transcriptomic Profiles of Two Human Leukemia Cells with Different Stemness
Source: Nanomaterials (Basel). 2020 Sep 30;10(10):1951. doi: 10.3390/nano10101951 (PMC7600526; doi:10.3390/nano10101951)
Supplement: Supplementary file 1 [file nanomaterials-10-01951-s001.pdf]

Supplementary information

Table S1. The qPCR primers sequences used in this study.

HL60		KG-1a	
**Name**	**Primer sequence (5' - 3')**	**Name**	**Primer sequence (5' - 3')**
ABCA1-F	AGGCTTGTCAAGGGGTAGGA	AMT-F	CGATGCAGAGGGCTGTAAGT
ABCA1-R	GCAGCAGCTGACATGTTTGT	AMT-R	GTACTGCACTGGCAGACTCC
ACACA-F	CTCTTGGCCTTTTCCCGGTC	ASMTL-F	CCATGGTGCTGTGCCCGGTGA
ACACA-R	GTTATCCCCAAACCCAGGCA	ASMTL-R	GCTCCAATGACCACGTCGGG
BAG6-F	GTCATCCGGATTTCCCACCA	CEP170-F	TTGCCGTTACCGCTATGTGT
BAG6-R	CATTGGTACCCAGACCGGC	CEP170-R	GCTACGTCATCCCCTTACCG
BTBD7-F	AGACGGAATGCCTGAGTGAC	FTH1-F	GCTTCAACAGTGCTTGGACG
BTBD7-R	TTTTGAGGGTGGCAAGACCA	FTH1-R	GTCCTGGTGGTAGTTCTGGC
ERMARD-F	TGCGCATGAGTATCAGCAGT	GNRHR-F	AACCAATGCCTTGGGCTTCT
ERMARD-R	CCCTCAACTCCACCCTGTCT	GNRHR-R	TCTATTGCTGCTATGCATACATTT
FADS2-F	CATGGGGAAGGGAGGGAACCA	POU1F1-F	TGTGATGTCTACAGCAACAGGA
FADS2-R	CACCAATCAGCAGGGGTTTCA	POU1F1-R	TGAAATCAGCAGCTGTGGGG
GBA-F	ACTTGCAGGGCTAACCTAGTG	SLC30A1-F	GGGATGGCTACGTTTTGGGA
GBA-R	AGCTTTTAGGGATGCAGGGG	SLC30A1-R	GCAGGTTATGGTTTTGGCCC
HVCN1-F	CGTGGTGGTGGTCTCATTCA	SLC6A20-F	TGAGGAGTGTGAGAAGGCGT
HVCN1-R	AGAAGTCCATGCTGTCGCAA	SLC6A20-R	GCTGCTCTATCTTGCCAGTGGACTC
LY6E-F	GGAGAGCCTCAGTCCCTGTA	TGFB2-F	CTCCGAAAATGCCATCCCGC
LY6E-R	ATTTGAGGCACCAAAGCAGC	TGFB2-R	AAGGAGAGCCATTCGCCTTC
PRPF31-F	ACAGTGGTGCGCGGAGAGGAGAGGC	TMEM236-F	TTAGGGGTTCCTTCAAAGGTGG
PRPF31-R	CAGGCGCGGCCTCCACTGGT	TMEM236-R	AGTACTGGCAATACAGCTAAGCC

Table S2. Mapping statistics of RNA-seq (each sample had two biological replicates).

Sample Name	Total reads	Total mapped reads	Total mapped rate (%)	Total uniq mapped reads	Total uniq mapped rate (%)	Total multiple mapped reads	Total multiple mapped rate (%)
KG1a-1	160812312	156531009	97.34	134973733	86.23	18213338	11.64
KG1a-2	152879934	148856987	97.37	128226490	86.14	17772043	11.94
KG1a/FeNPs-1	165554692	160836048	97.15	139459188	86.71	17574442	10.93
KG1a/FeNPs-2	179570162	175131734	97.53	151893700	86.73	20378326	11.64
KG1a/PBNPs-1	172683280	167773215	97.16	145767341	86.88	18481010	11.02
KG1a/PBNPs-2	181736920	176630722	97.19	155424108	87.99	18282206	10.35
HL60-1	172758326	168502943	97.54	149451241	88.69	15600168	9.26
HL60-2	184046760	179242559	97.39	156951758	87.56	18620171	10.39
HL60/FeNPs-1	193393098	188522987	97.48	164922610	87.48	19700413	10.45
HL60/FeNPs-2	181866538	177124798	97.39	156482333	88.35	17283987	9.76
HL60/PBNPs-1	191944518	187215996	97.54	165411934	88.35	18252962	9.75
HL60/PBNPs-2	157784478	154041579	97.63	137079316	88.99	14710873	9.55

Table S3. The mRNA peak insert size in all samples.

Samples	Peak Insert Size (bp)
HL60-1	317.10
HL60-2	322.71
HL60/FeNPs-1	313.19
HL60/FeNPs-2	318.04
HL60/PBNPs-1	314.78
HL60/PBNPs-2	317.50
KG1a-1	303.25
KG1a-2	300.49
KG1a/FeNPs-1	307.43
KG1a/FeNPs-2	309.94
KG1a/PBNPs-1	314.14
KG1a/PBNPs-2	324.19

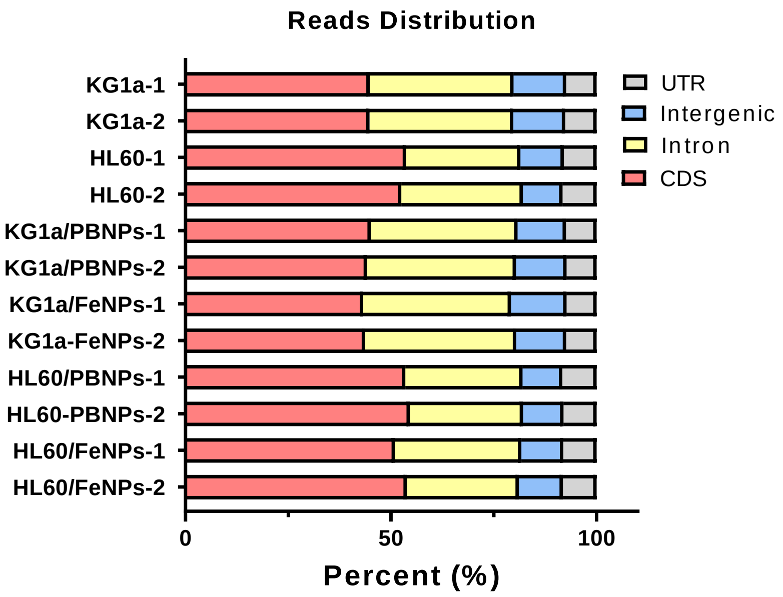

Figure S1. Statistics of the distribution of Reads in different regions of the genome. UTR: Untranslated Regions; Intergenic: intergenic region; Intron: intron region; CDS: coding region.

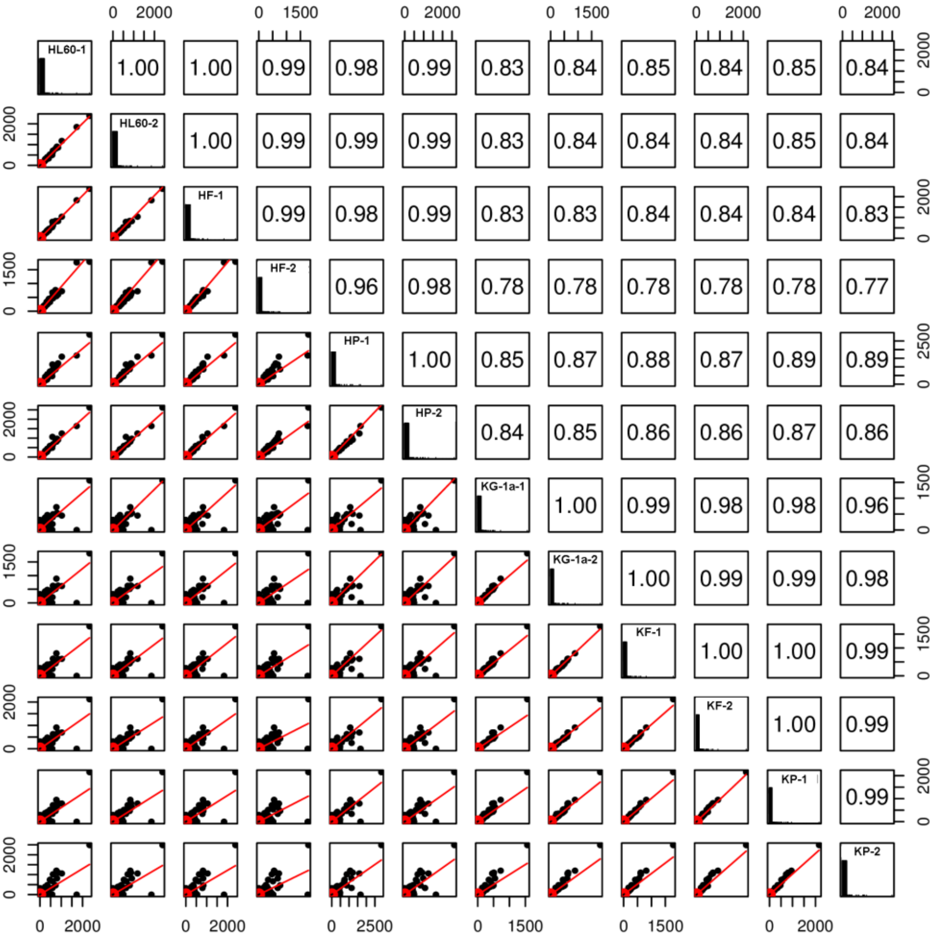

Figure S2. Correlation analysis of gene expression in 12 samples. The horizontal or vertical coordinates of each small square were the two samples for comparison, and the numbers in the squares were the Pearson correlation coefficients (HF: HL60-FeNPs, HP: HL60-PBNPs, KF: KG1a-FeNPs, KP: KG1a-PBNPs).

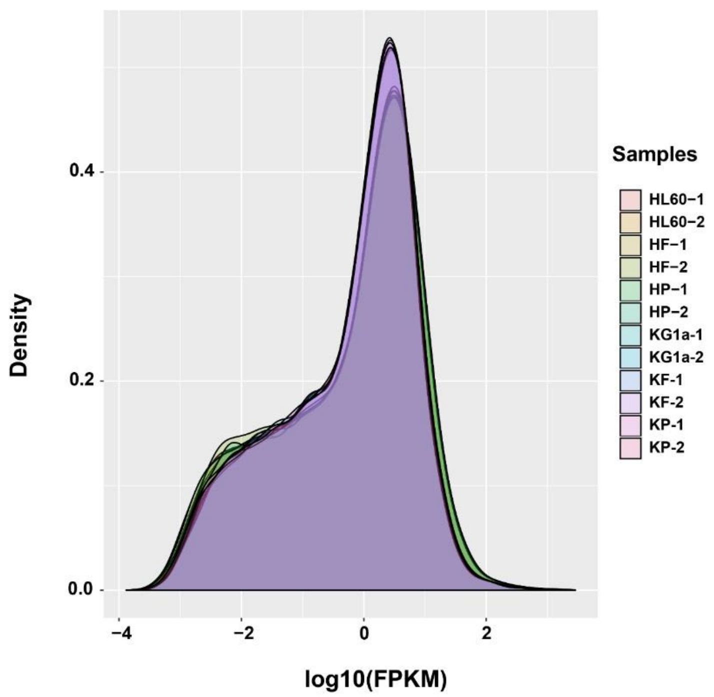

Figure S3. Density map of gene expression level of all samples. HF: HL60-FeNPs, HP: HL60-PBNPs, KF: KG1a-FeNPs, KP: KG1a-PBNPs). Each sample had two biological replicates.

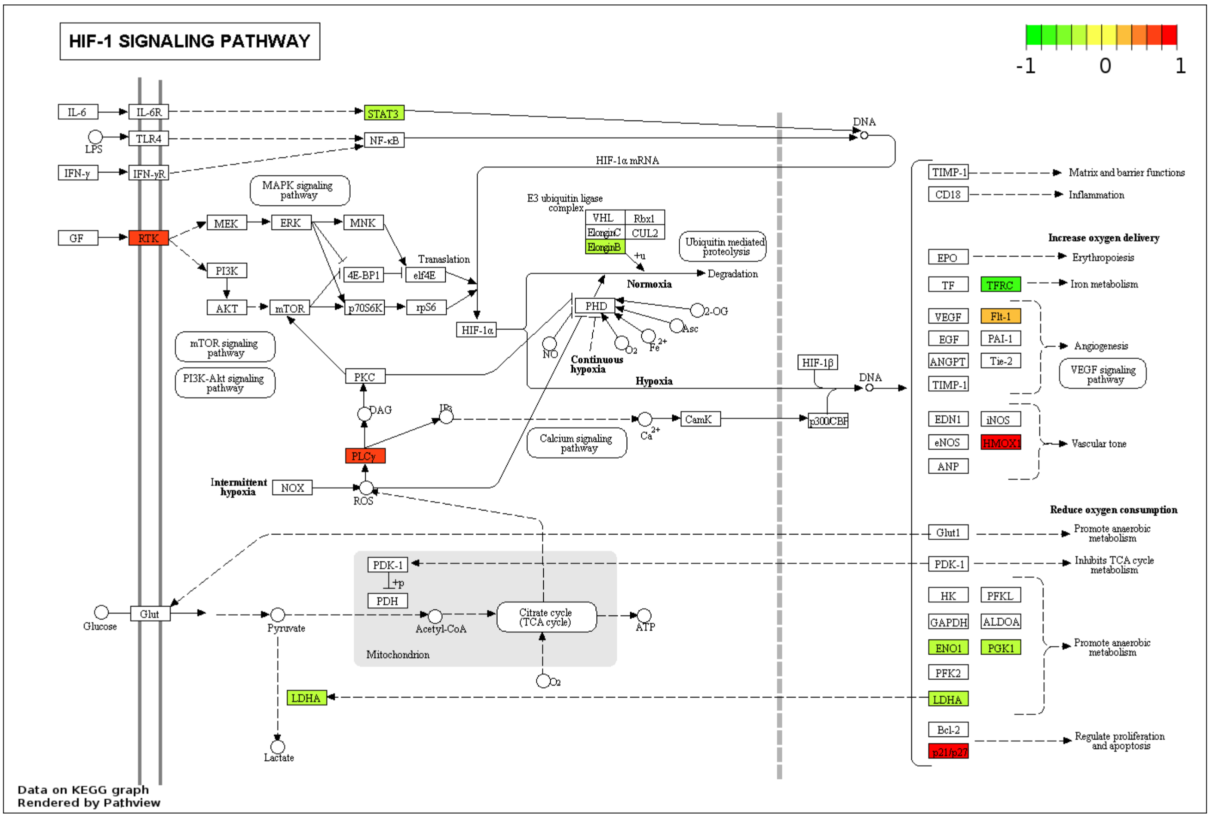

Figure S4. KEGG analysis of the HIF-1 signaling pathway in Pathview. The up-regulated genes were labelled by red, down-regulated genes were colored by green.

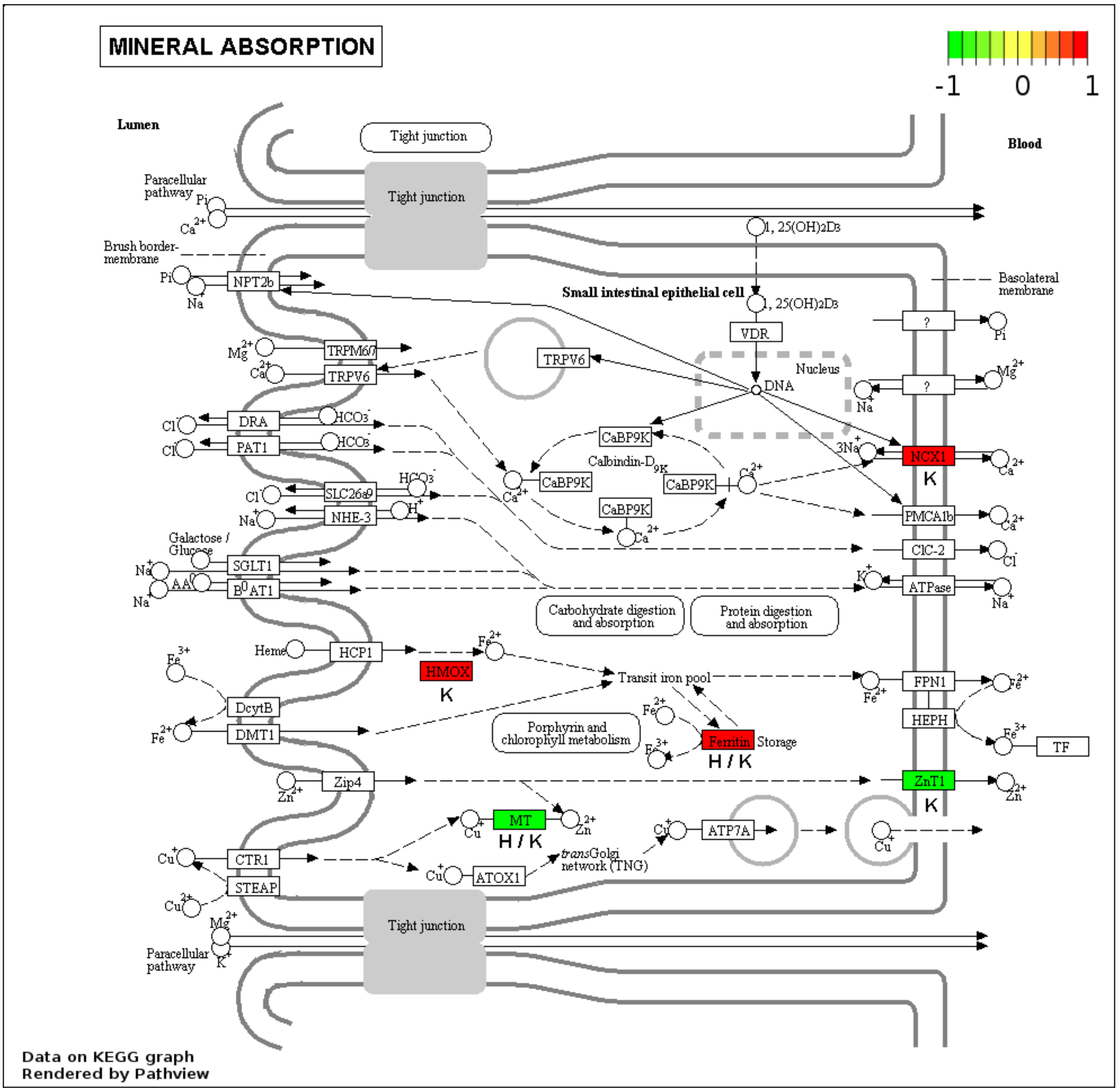

Figure S5. KEGG analysis of the Mineral absorption pathway in Pathview. The up-regulated genes were labelled by red, down-regulated genes were colored by green.
